# The power of innate: Behavioural attachment and neural activity in responses to natural and artificial objects in filial imprinting in chicks

**DOI:** 10.3389/fphys.2022.1006463

**Published:** 2022-11-21

**Authors:** A. B. Cherepov, A. A. Tiunova, K. V. Anokhin

**Affiliations:** ^1^ Institute of General Pathology and Pathophysiology, Moscow, Russia; ^2^ P. K. Anokhin Institute of Normal Physiology, Moscow, Russia; ^3^ Institute for Advanced Brain Studies, Lomonosov Moscow State University, Moscow, Russia

**Keywords:** imprinting, domestic chick, learning, innate predisposition, *c-fos* expression

## Abstract

Newly hatched domestic chicks are known to orient preferentially toward naturalistic stimuli, resembling a conspecific. Here, we examined to what extent this behavioral preference can be transcended by an artificial imprinting stimulus in both short-term and long-term tests. We also compared the expression maps of the plasticity-associated *c-fos* gene in the brains of chicks imprinted to naturalistic (rotating stuffed jungle fowl) and artificial (rotating illuminated red box) stimuli. During training, the approach activity of chicks to a naturalistic object was always higher than that to an artificial object. However, the induction of *c-fos* mRNA was significantly higher in chicks imprinted to a box than to a fowl, especially in the intermediate medial mesopallium, hyperpallium apicale, arcopallium, and hippocampus. Initially, in the short-term test (10 min after the end of training), chicks had a higher preference for a red box than for a stuffed fowl. However, in the long-term test (24 h after imprinting), the response to an artificial object decreased to the level of preference for a naturalistic object. Our results thus show that despite the artificial object causing a stronger *c-fos* novelty response and higher behavioral attachment in the short term, this preference was less stable and fades away, being overtaken by a more stable innate predisposition to the naturalistic social object.

## Introduction

Newly hatched chicks of precocial birds rapidly form strong preference for an appropriate object that they encounter early in life. Several species, including domestic chicks (*Gallus gallus domesticus*), orient preferentially toward naturalistic stimuli, resembling a conspecific in such critical aspects as size, complexity, motion pattern, and specific visual details (Bolhuis et al., 1985; Johnson and Horn, 1988; Bolhuis, 1999; Vallortigara et al., 2005; Rosa-Salva et al., 2019). Once developed, the preference for a naturalistic stimulus is difficult to be reversed by exposure to a less naturalistic stimulus (Bolhuis and Trooster, 1988). Moreover, the stability of the preference for a naturalistic object is further enhanced by a separate process of innate predisposition development (Bolhuis et al., 1985). However, the interaction of this developmental process with imprinting and their comparative neural bases are insufficiently understood. The aim of the present study was to examine short- and long-term stimulus preference of newborn chicks after exposure to an artificial object followed by a naturalistic object and vice versa. We also studied patterns of brain responses in the chicks imprinted to naturalistic and artificial stimuli or re-exposed to the previously imprinted stimulus versus exposed to a novel stimulus. To this end, we used *in situ* hybridization analysis of *c-fos* immediate-early gene expression, known to be induced by neuronal activation (Minatohara et al., 2016), including formation and retrieval of memory in the chick brain (McCabe and Horn, 1994; Abramova and Anokhin, 1998; Suge and McCabe, 2004; Suge et al., 2010; Yamaguchi et al., 2010; Tiunova et al., 2019).

## Materials and methods

Chicks of both sexes at the age of 24–72 h were used in the experiments. Chicken embryos of the Lohmann Brown strain were obtained from a local supplier on E12-E15 and incubated in darkness. After hatching, they were maintained in darkness in individual boxes. At the age of 24 ± 8 h, the chicks were placed individually in a running wheel and exposed to an imprinting object (a rotating stuffed jungle fowl or rotating illuminated red box). Species-specific maternal calls were played back during the training. The number of wheel revolutions toward the training stimulus and in the opposite direction was recorded.

**FIGURE 1:**
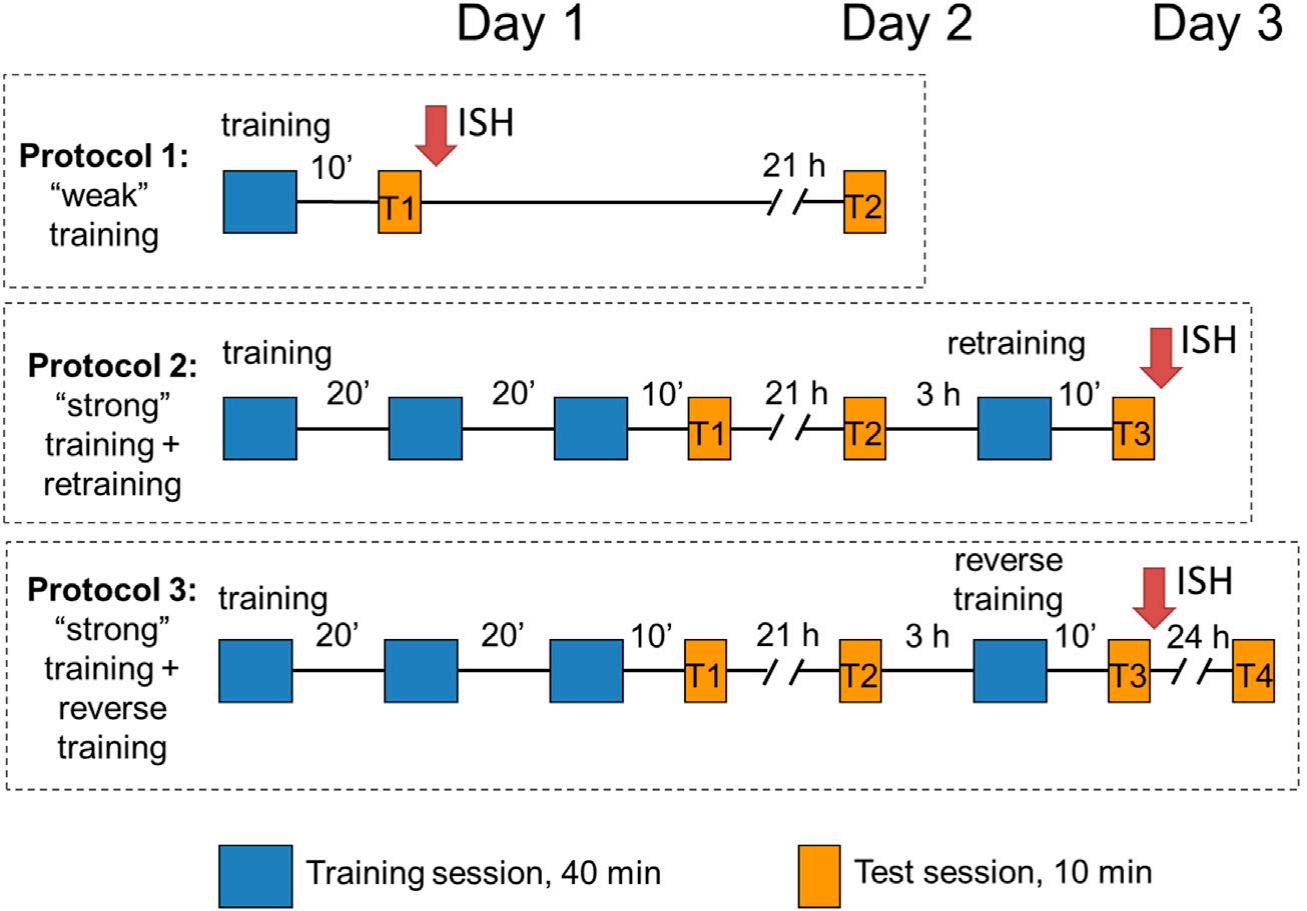
Design of the experiments.

Three protocols were used in the study (Figure 1). In protocol 1 (“weak” training), chicks were exposed to imprinting stimulus for 40 min and tested after a 10-min pause in a 10-min sequential preference test (see below). Chicks in the other two protocols (protocols 2 and 3) received three consecutive 40-min imprinting sessions separated by 20-min breaks (“strong” training), tested for the object preference after a 10-min pause (test 1) and returned to their dark boxes. Following 21 h, they were tested again (test 2), and 3 h later (i.e., at the age of 48 ± 8 h), they were exposed for 40 min to either a familiar object (protocol 2: re-training) or an unfamiliar object (protocol 3: reverse training). Then, 10 minutes later, they were tested once more (test 3). In addition, some of the chicks from protocol 3 (reverse training) were returned to home boxes and tested 24 h later (test 4). Thus, tests 1 and 3 were performed to evaluate the short-term memory after the “weak” or “strong” training, respectively, and tests 2 and 4 were conducted to evaluate the long-term memory.

For the preference test, chicks were placed into the same running wheels and presented sequentially with the stuffed fowl and red box in the following order: familiar–unfamiliar–unfamiliar–familiar. Each object was demonstrated for 2 min with a 40-s pause between them. The objects were illuminated in the same manner as during training; however, the maternal call was not played back (McCabe et al., 1981). To exclude potential confounding effects of motor activity on the results of preference tests, the chicks that made less than four wheel rotations were excluded from the analysis and further procedures. In each test, a preference score (in %) was calculated for each bird as (number of the wheel rotations toward the training stimulus X 100)/(total number of the rotations). The differences between groups were analyzed using ANOVA, the *post hoc* Tukey HSD test, and *t*-test in Statistica 6.0. Immediately after tests 1 and 3, some of the chicks were sacrificed and their brains were frozen for *in situ* hybridization analysis. Then, 15-µm cryostat brain sections were collected throughout the whole brain with 200-µm intervals, and *c-fos* mRNA was detected by radioactive *in situ* hybridization, as described elsewhere (Anokhin et al., 1991). Briefly, the sections were incubated for 12 h at 38°C with the synthetic single-stranded oligodeoxynucleotides corresponding to 2,341–2,383 bases of the chicken *c-fos* gene (Fujiwara et al., 1987), labeled with [^33^P]-dATP (>148 PBq/mol) using terminal deoxynucleotidyl transferase (Boehringer Mannheim). A sense oligodeoxynucleotide of the same length was used as a negative control. After hybridization and washing, sections were dehydrated and exposed to a hyperfilm *β*-max x-ray film (Amersham) for 3–4 days along with the ^14^C plastic radioactivity standards (Amersham). Resulting films were digitized using a flatbed scanner, and the optic density in selected brain regions was measured using ^33^P-converted (Eakin et al., 1994) calibration curves plotted using eight standard points. The probe concentration and the exposure time were determined in pilot experiments to stay within the linear part of the calibration curve. Since the induction of *c-fos* expression in the brain was massive and widespread, the background correction was performed by normalizing the images against the blank space between sections in the slides. The optical density of the radioactive signal was measured in the selected brain areas (see Supplementary Figure S1) defined by the chick brain atlas (Kuenzel and Masson, 1988), and quantitative analysis was performed by ImagePro Plus 3.0 image analysis software (Media Cybernetics). mRNA levels were measured in all brain sections that contained a given structure (4–8 sections per brain structure), and the average for each structure was calculated and used for further statistics. Between-group differences were estimated using one-way ANOVA, factorial ANOVA, *post hoc* Tukey HSD, and unequal N HSD tests in Statistica 6.0.

In total, 125 chicks were used in the experiments (see Supplementary Table S1 for the group numbers). The study was carried out in accordance with the recommendations of the Directive 2010/63/EU of the European Parliament and the Council of the European Union issued on 22 September 2010, on the protection of animals used for scientific purposes (Section 27). The protocol was approved by the Animal Ethics Committee of the P.K.Anokhin Research Institute of Normal Physiology.

## Results

### Approach activity

**FIGURE 2:**
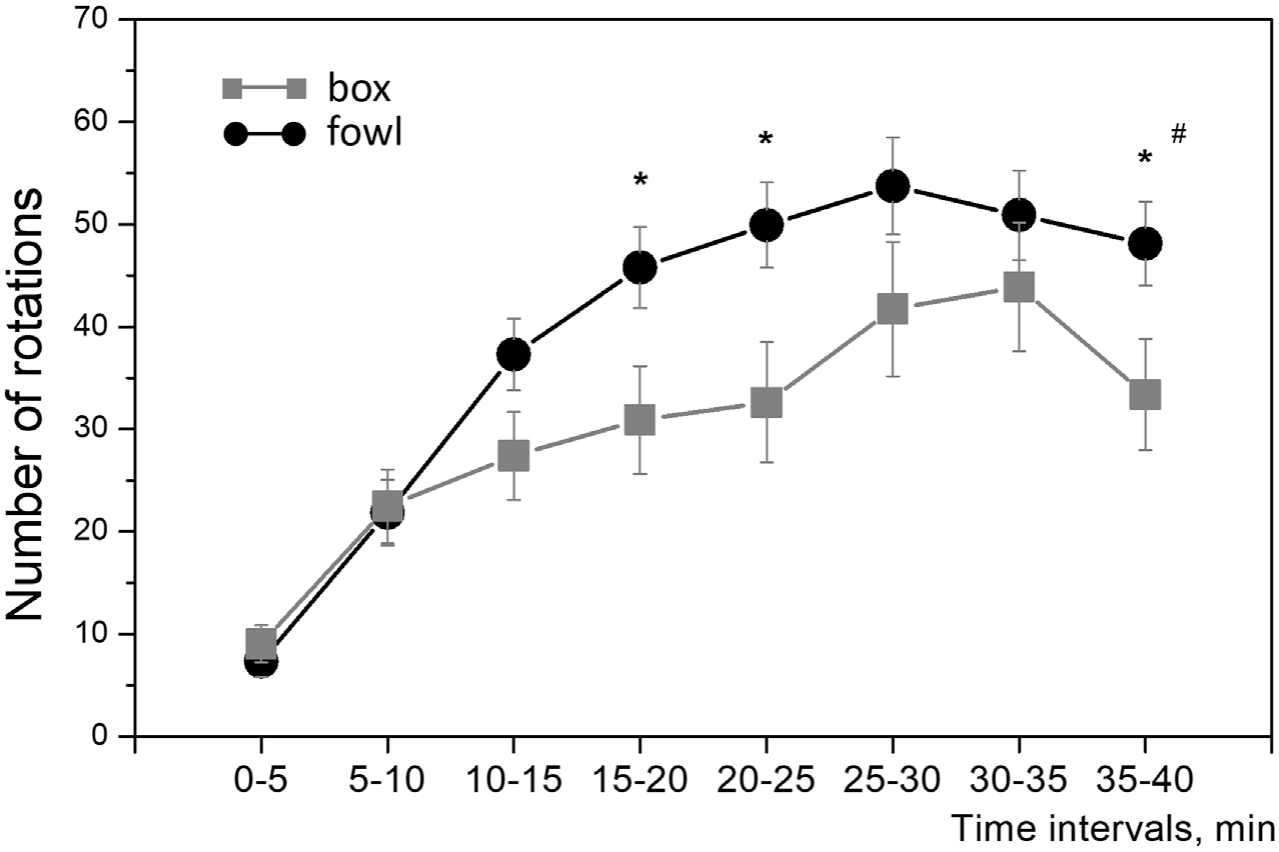
Mean number of the running wheel revolutions toward the imprinting stimulus during the 40-min session. Each point represents a 5-min time interval. ^#^ *p* <0.01 between groups (one-way ANOVA); *p <0.05 between groups at the 5-min interval (*post hoc* Tukey HSD test).

**FIGURE 3:**
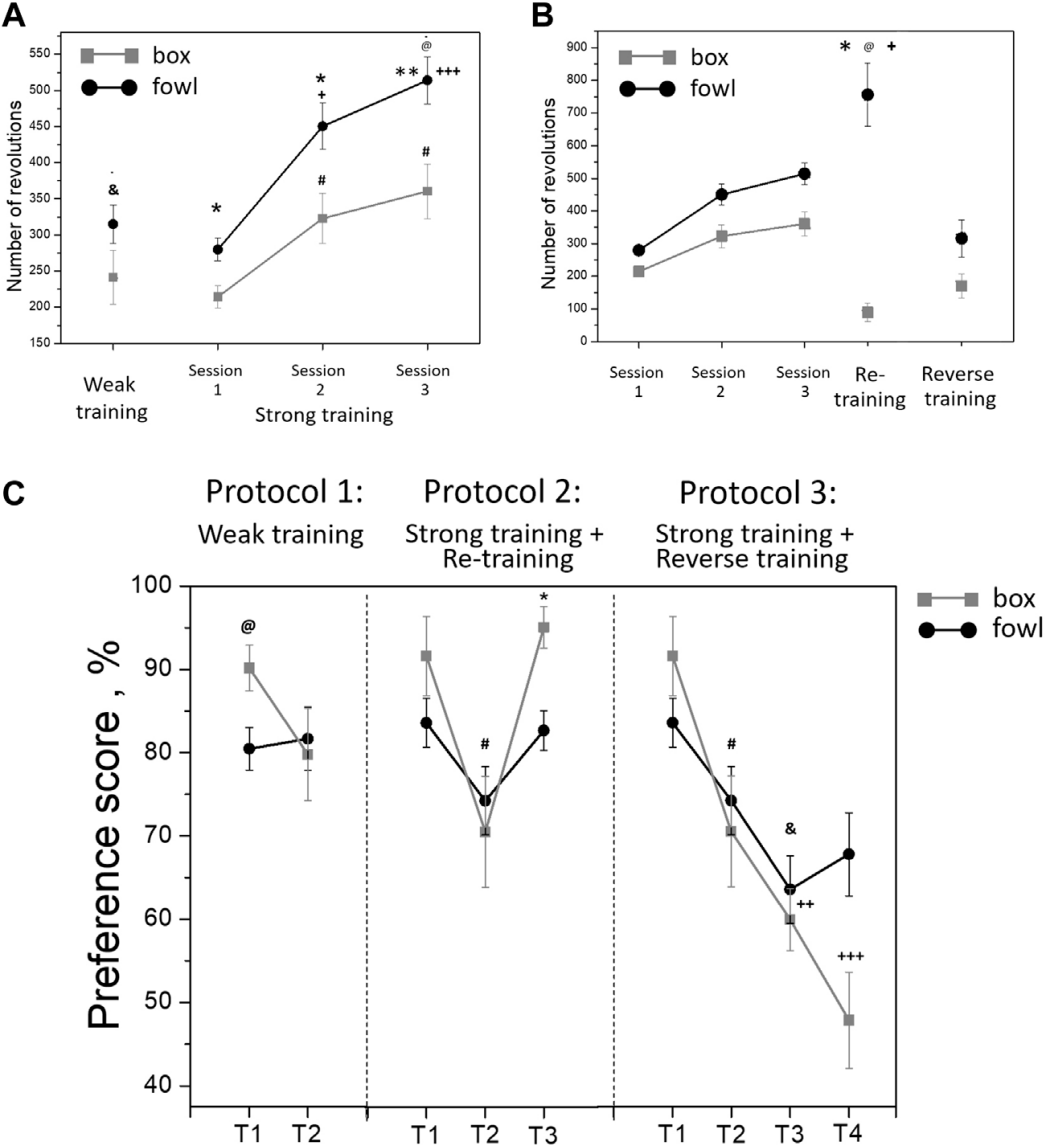
**(A)** Mean number of the running wheel revolutions toward the imprinting stimulus during a single 40-min session (weak training, ^&^ *p* <0 .01 between box-exposed and fowl-exposed chicks, *t*-test) and three 40-min sessions (strong training). ^@^ *p* <0.001 between box-exposed and fowl-exposed chicks (factorial ANOVA and stimulus type X session); *p <0.05 and **p <0.01 between box-exposed and fowl-exposed chicks (*post hoc* Tukey HSD test), respectively; ^+^ *p* <0.05 and ^+++^ *p* <0.001 as compared with session 1 (hen-exposed); ^#^ *p* <0.001 as compared with session 1 (box-exposed). **(B)** Mean number of the running wheel revolutions toward the imprinting stimulus during imprinting (strong training), exposure of the imprinted chicks to a novel stimulus (reverse training; *n* = 16) or re-exposure to the familiar stimulus (re-training, *n* = 19). ^@^ *p* <0.001 between box-exposed and fowl-exposed chicks (factorial ANOVA and stimulus type X session); *p <0.01 between session 3 and re-training in the fowl-exposed chicks; ^+^ *p* <0.001 between reverse training and re-training in the fowl-exposed chicks (*post hoc* Tukey HSD test). **(C)** Preference score of the chicks after weak training (T1—10 min after the training, T2—24 h after the training; *n* = 12 per group); after strong training and reverse training (T1—10 min after the training, T2—24 h after the training, T3—10 min after the reverse training, and T4—24 h after the reverse training); after strong training and re-training (T1—10 min after the training, T2—24 h after the training, and T3—10 min after the re-training). ^@^ *p* <0.05 between box-exposed and fowl-exposed chicks (one-way ANOVA); ^#^ *p* <0.05 between T1 and T2 in the box-exposed chicks (*post hoc* unequal N HSD test); ^&^ *p* <0.05 between T1 and T3 in the fowl-exposed chicks; ^++^ *p* <0.01 between T1 and T3 in the box-exposed chicks; ^+++^ *p* <0.001 between T1 and T4 in the box-exposed chicks; *p <0.001 between T2 and T3 in the box-exposed chicks (*post hoc* Tukey HSD test).

During training, the approach activity of chicks to the naturalistic object was always higher than that to the artificial one. Within the single 40-min session in protocol 1 (“weak” training), the number of wheel rotations increased steadily in both groups (Figure 2). The running activity was significantly higher in the fowl-exposed chicks than that in the box-exposed chicks both throughout the entire session and for total count (one-way ANOVA: F = 8.88; *p* = 0.0033; Figure 2). Similarly, the activity of chicks trained in protocols 2 and 3 (“strong” training) steadily increased from session to session and was significantly higher in the chicks exposed to the fowl (Figure 3A). Factorial ANOVA analysis revealed significant influence of the stimulus factor (F = 31.4, *p* <0.001) and session factor (F = 40.2, *p* <0.001). No significant factor interaction was found (F = 2.0, *p* >0.05).

During re-training (protocol 2) and reverse training (protocol 3), the activity of box-exposed chicks was significantly different from the fowl-exposed chicks. Factorial ANOVA analysis revealed significant influence of the stimulus factor (F = 59.5, *p* <0.001) and session factor (F = 20.3, *p* <0.001). The interaction of the stimulus and session factors was also significant (F = 6.4, *p* <0.001). Re-exposure to the naturalistic stimulus (the stuffed fowl) 24 h later induced a high approach activity, the number of wheel rotations toward the stimulus being significantly greater than during the last training session (Figure 3B; *p* <0.01, *post hoc* Tukey HSD test). In contrast, activity during re-exposure to the artificial stimulus (box) decreased compared to the last training session and was significantly lower than that in the chicks re-exposed to the stuffed fowl (Figure 3B; *p* <0.001, *post hoc* Tukey HSD test). The activity during reverse training (protocol 3) was significantly lower in both fowl-imprinted and box-imprinted chicks than the last training session (Figure 3B; *p* <0.001, *post hoc* Tukey HSD test). The number of rotations toward the naturalistic stimulus was significantly higher in chicks that were re-exposed to it than that in the chicks that were exposed to the fowl after being trained to the box (Figure 3B; *p* <0.001, *post hoc* Tukey HSD test).

### Preference score

In the short-term test in protocol 1 (T1, 10 min after the end of “weak” training), the preference score was higher in the box-imprinted chicks (one-way ANOVA: F = 6.55, *p* <0.05; Figure 3C). However, in the long-term test (T2, 21 h later), preference for the box in these chicks decreased, while the preference for the fowl remained stable. Factorial ANOVA analysis (stimulus-type X test session) in case of “weak” training revealed the effect of the test session only at a tendency level (F = 3.6, *p* = 0.062). In both short-term and long-term tests, the motor activity of chicks did not differ between the groups (T1: trained to the box—29.9 wheel revolutions, trained to the fowl—37.1; T2: trained to the box—27 revolutions, trained to the fowl—22.4, *p* >0.05, factorial ANOVA). Similarly, the T1 short-term test after the “strong” training (protocols 2 and 3) demonstrated higher preference in the box-imprinted chicks, but this preference significantly decreased in the T2 long-term test (Figure 3C, protocols 2 and 3, T1, T2, F = 11.3, *p* <0.01), and there was no difference between the box-imprinted and fowl-imprinted chicks in this long long-term test. However, in contrast to the weak training, the motor activity during the tests differed between the groups. In both short-term and long-term tests, the total number of the wheel revolutions in the chicks strongly imprinted to the fowl was higher than that in the box-imprinted chicks (T1: trained to the box—21.6 wheel revolutions, trained to the fowl - 38.4, *p* <0.05; T2: trained to the box—26.9 revolutions, trained to the fowl—61.4, *p* <0 .05). Factorial ANOVA analysis (type of stimulus X test session) showed effects of the stimulus (F = 9.7, *p* <0.01) and effects of the session (F = 4.3, *p* <0.01). No interaction of the factors was found.

Chicks re-exposed to the box (protocol 2: re-training) demonstrated high preference in the T3 test (*p* <0.001, *post hoc* Tukey HSD test compared with T2 test, while the preference score in chicks re-exposed to the fowl remained at the same level (Figure 3C, protocol 2, T1-T3). The motor activity during the test did not differ between the groups. The total number of the wheel revolutions was 56.8 in the chicks re-exposed to the box and 80 in those re-exposed to the fowl (one-way ANOVA, F = 1.2, *p* >0.05).

After the reverse training, both fowl-imprinted and box-imprinted chicks showed decrease of the preference in the T3 short-term test (Figure 3C, protocol 3, T3). Factorial ANOVA analysis revealed significant influence of the test factor (F = 13.7, *p* <0.001). No significant effect of the stimulus was found, and the factor interaction effect was observed at the tendency level (F = 2.2, *p* = 0.09). However, in the T4 long-term test, chicks imprinted to the stuffed fowl and then exposed to the box demonstrated preference for the fowl (67%), while the chicks imprinted to the box and then exposed to the fowl showed no preference (48%). Preference for the box in the chicks imprinted to the box and then exposed to the fowl was significantly lower in both short-term and long-term tests (T3 and T4, respectively) compared with the first test (T1) (*p* <0.01 for T3, *p* <0.001 for T4, Tukey HSD test). On the other hand, the reverse training in the chicks imprinted to the fowl and then exposed to the box produced only a short-term effect. Their preference to the fowl decreased as a result of exposure to the box only in the following T3 test (*p* <0.05, Tukey HSD test). Factorial ANOVA analysis revealed no significant differences in the total number of the wheel revolutions between the groups and between the test sessions (imprinted to the box and exposed to the fowl: 99.8 in the T3 test and 71.2 in the T4 test; imprinted to the fowl and exposed to the box: 96.3 in the T3 test and 89 in the T4 test; *p* >0.05).

Furthermore, analysis revealed the effect of novelty of the stimulus on the preference score to the same stimulus (Protocol 2: re-training) or to a novel stimulus (Protocol 3: reverse training) in the short-term test after the second training. Factorial ANOVA showed significantly lower preference in the chicks trained to a novel stimulus than those re-trained to the familiar stimulus (F = 40.4, *p* <0.001). No significant effect of the stimulus type was observed.

### Training-induced *c-fos* expression

**FIGURE 4:**
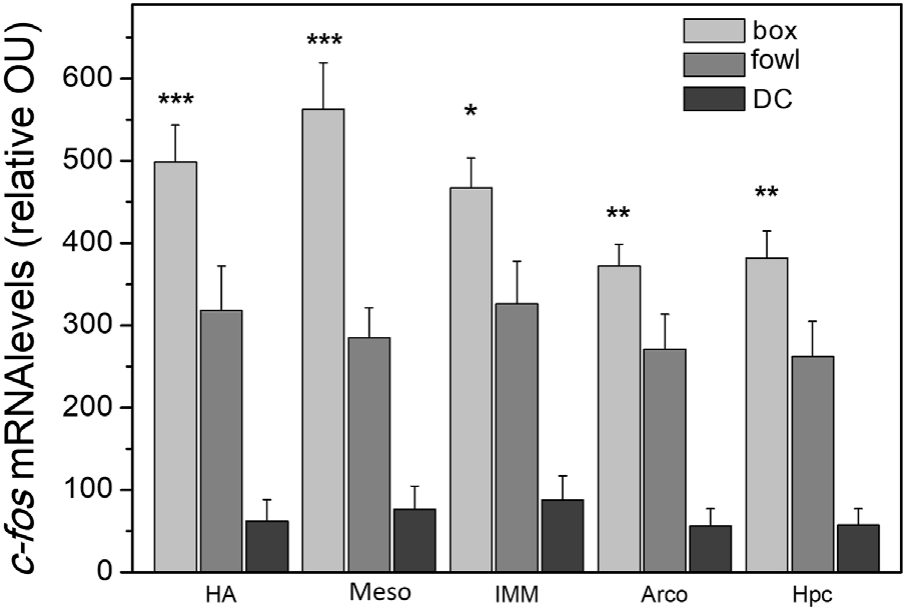
*c-fos* mRNA levels in the brain areas of chicks after the 40-min imprinting session. *n* = 6 (box), *n* = 9 (fowl), and *n* = 5 (dark control, DC). (factorial ANOVA, stimulus type X training condition, and *post hoc* unequal N HSD test); *p <0.05, **p <0.01, and ***p <0.001.

A single 40-min training session (protocol 1) produced massive widespread induction of *c-fos* mRNA in the chick brain. In all the structures examined, the level of *c-fos* mRNA was significantly higher in the brains of imprinted chicks than that in the dark control (Supplementary Figure S1; Figure 4). Additionally, in several brain regions, *c-fos* expression was higher in the box-imprinted chicks than that in the fowl-imprinted chicks (Figure 4, see below for statistics).

Re-exposure of chicks to a familiar object 24 h after the initial training (protocol 2) also caused widespread *c-fos* mRNA induction. No differences in the expression level were found between the chicks re-exposed to the stuffed fowl and to the box.

Reverse training (protocol 3) also produced substantial elevation of *c-fos* mRNA levels in both groups (fowl-box- and box-fowl-exposed chicks) compared with the dark control; no difference was found between the object-exposed groups.

To examine *c-fos* induction in response to different stimuli and types of behavioral treatments assuming the possibility of independent regulation of its expression in different brain areas, we analyzed *c-fos* mRNA changes in each brain region separately. Analysis was performed using factorial ANOVA (type of stimulus X training condition), the training conditions being protocol 1 (“weak training”), protocol 2 (re-exposure to the same stimulus after “strong training”), and protocol 3 (exposure to a novel stimulus after “strong training”). We found a significant stimulus effect in the hyperpallium apicale (HA: F = 6.6, *p* <0.05), medial mesopallium (Meso: F = 12.3, *p* <0.001), intermediate medial mesopallium (IMM: F = 7.6, *p* <0.05), arcopallium (Arco: F = 15.0, *p* <0.001), and hippocampus (Hpc: F = 16.2, *p* <0.001). The effect of the factor interaction was revealed in the arcopallium (F = 8.8, *p* <0.01) and medial mesopallium (F = 3.2, *p* <0.05). *Post hoc* comparisons showed higher expression in several brain areas of the box-imprinted chicks than in the fowl-imprinted chicks: HA (*p* <0.001), Meso (*p* <0.001), IMM (*p* <0.05), Arco (*p* <0.01), and hippocampus (*p* <0.01) (Figure 4).

## Discussion

The first objective of the present study was to examine behavioral effects of the weak and strong training protocols in chicks imprinted on either an artificial or a naturalistic stimulus. We found that the approach activity of chicks increased both during and between the training sessions (Figures 1, 3A). The approach activity to the naturalistic stimulus (stuffed fowl) was higher than the activity toward the artificial stimulus (box) throughout the whole training experiment. This is consistent with the evidence that naturalistic objects are particularly effective in stimulating the approach (Bolhuis et al., 1985; Bolhuis, 1991; Rosa-Salva et al., 2019). A sequential test with two objects conducted 10 min after the end of the weak or strong training demonstrated higher preference for the stimulus that was used during the training. Interestingly, the level of preference was higher in the chicks weakly imprinted to the box than that in those imprinted to the stuffed fowl (Figure 3C, T1). In the test given 24 h after weak training, however, the preference in the box-imprinted chicks decreased, while in the fowl-imprinted chicks, it remained stable (Figure 3C, T2). Factorial ANOVA analysis of the preference score with the stimulus type and test session as factors revealed significant effects of the test session, and the *post hoc* analysis showed difference between T1 and T2 tests in box-imprinted chicks. This result matches the data that chicks imprinted to either a naturalistic or an artificial object gradually shift their preference to the naturalistic object (Bolhuis et al., 1985). Such an effect may be a manifestation of the development of an innate predisposition to a naturalistic stimulus—the process that is physiologically and behaviorally independent of the learning mechanisms (Horn and Johnson, 1989; Bolhuis and Honey, 1998). This developmental process may be further boosted by the chick’s own activity, such as running in the wheel (Bolhuis et al., 1985). Another mechanism that could contribute to the preference shift in box-imprinted chicks might be a transient preference for unfamiliar stimuli observed in the young chicks and explained by their tendency to explore novelty (Bateson and Jaeckel, 1976; Lemaire et al., 2021). However, in our experiments, there was no corresponding preference shift in the fowl-imprinted chicks; their preference level remained stable in both short-term and long-term tests. This result favors the hypothesis of the primary role of innate predisposition development in modulating the initially acquired preference for an artificial object. As a recent study has shown, innate predispositions were able to change learned preferences until these memories are fully consolidated (Lemaire et al., 2021).

The motor activity of chicks in tests given after weak training did not depend on the stimulus type and did not differ between the short-term (T1) and long-term (T2) tests. However, after strong training, the motor activity in both fowl-imprinted and box-imprinted chicks increased in the long-term test compared with the short-term test. Additionally, following strong training, the total number of wheel revolutions in short-term and long-term tests was higher in fowl-imprinted chicks than that in box-imprinted chicks. This difference might be due to the effect of intensive activity during training, particularly exposure to a naturalistic stimulus on the motivation and arousal levels of these chicks in the subsequent test sessions. There was no direct connection between the level of motor activity during the tests and preference score after strong training: while the total number of wheel revolutions increased from T1 to T2, the preference score declined (Figure 3C).

Next, we examined the effects of a second training either to the same or a novel stimulus (re-training and reverse training, respectively) given 24 h after the initial strong training. We found that chicks re-exposed to the fowl showed increased approach to it compared with the response after the initial training. On the contrary, the activity of chicks re-exposed to the box dropped dramatically (Figure 3B, re-training). This decrease in approaching the already imprinted stimulus may reflect a predisposition-driven shift to a naturalistic object that developed between the first and second training, and which was further facilitated by the chick’s wheel running activity during the first training and test sessions (Bolhuis et al., 1985). However, in the test T3 given 10 min after re-training, the preference score in the box-retrained chicks reached the maximal level which seems to contradict this explanation (Figure 3C, T3 in re-training). Thus, the second training to the artificial stimulus failed to produce a high approach activity toward it but nevertheless resulted in increased preference of this stimulus in the following test. Notably, the preference score in chicks imprinted to the fowl did not undergo such changes after repeated training. No differences in the motor activity of the chicks during the tests after second training were found. Taken together, these behavioral results show that preference for the naturalistic object remained stable in the short-term and long-term tests, as well as after the repeated training, while the preference formed for an artificial stimulus varied as a function of time and repeated training. The results of the reverse training support this conclusion (Figure 3C, reverse training). In the chicks previously imprinted to the fowl, exposure to the box did not affect the initially formed preference in the following short-term and long-term tests. In contrast, exposure to the fowl significantly reduced the preference for the previously learned artificial object in box-imprinted chicks (Figure 3C, T3 and T4 in reverse training).

Analysis of *c-fos* mRNA levels after 40-min training revealed massive induction of expression in all the examined brain regions (Figure 4 and Supplementary Figure S1). Despite the lack of precision inherent to radioactive *in situ* hybridization and multiple factors that complicate standardization procedures (e.g., the need for comparable labeling of antisense and sense probes, correction for the background of the x-ray film, matching the thickness of radioactivity standards and tissue sections, and ensuring that hybridization signals do not fall within the saturation part of the calibration curve) (Le Moine, 2000; Chen et al., 2012), the large *c-fos* mRNA expression in response to imprinting stimuli allowed to identify the most prominent regions of the chick brain that participated in this reaction. These included tectofugal and thalamofugal visual projection regions [the entopallium, tectum opticum, HA, and hyperpallium densocellulare (HD)], regions involved in different aspects of imprinting (the IMM, mediorostral nidopallium/mesopallium (MNM), medial striatum (MSt), HD, and hippocampus), and other areas (the medial mesopallium, medial nidopallium, arcopallium, and cerebellum). In most structures, the *c-fos* mRNA level was higher in chicks imprinted to the box than that to the fowl, and in several structures, this difference reached statistical significance (Figure 4). Since the locomotor activity during training was even higher in the box-exposed chicks, the motor activity in the T1 test was comparable and all other parameters were equalized between the groups, we conclude that a higher level of expression reflected the higher degree of “novelty” of the artificial stimulus, i.e., its lesser similarity to the innate model. This finding corresponds well with the evidence for the role of novelty in the induction of *c-fos* in the mammalian and avian brain (Anokhin and Sudakov, 1993; Zhu et al., 1995; Perez et al., 2020).

The structures where we observed this difference included IMM, a region critical for preference acquisition, in which *c-fos* expression can be induced by both training and retrieval of imprinting memory (McCabe and Horn, 1994; Tiunova et al., 2019). Induction of *c-fos* protein expression was also found in the IMM of chicks that made a spontaneous choice between a more “artificial” stimulus (scrambled fowl) and a “natural” stimulus (stuffed fowl) (Mayer et al., 2016). In this study, chicks were primed, first, by non-species-specific acoustic stimulation and then by light in a featureless environment, after which they were given a choice between the “artificial” and “natural” stimuli. Most of the chicks chose the stuffed fowl which corresponds well with the fact that the non-species-specific acoustic stimulation primes development of innate template-based predisposition to prefer a naturally looking object (Egorova and Anokhin, 2003). However, in the IMM of chicks that preferred the scrambled fowl, the level of *c-fos* expression was higher than in those that preferred the stuffed fowl (Mayer et al., 2016). The authors concluded that the greater difference between the innate template and the “artificial” stimulus required greater neuronal plasticity which was reflected in the greater *c-fos* expression. Our present results suggest that learning of the artificial object is also accompanied by a more pronounced *c-fos* expression than of the natural object.

Other areas where the expression was higher in the box-exposed chicks included the HA, arcopallium, medial mesopallium, and hippocampus, the structures connected with the IMM and involved, in different ways, in the imprinting and social cohesion (Bradley et al., 1985; Suge and McCabe, 2004; Bock et al., 2005; Maekawa et al., 2006; Aoki et al., 2015; Mayer et al., 2019). Interestingly, in the study cited previously (Mayer et al., 2016), no differences in *c-fos* expression were found in the HA. The induction of *c-fos* in the HA in our experiments may reflect its dissimilar role in manifestation of an innate predisposition and in memorizing the learned stimulus.

Taken together, our results show that although an artificial object causes a stronger novelty brain response and higher behavioral attachment in the short term, this preference is less stable and fades away, being overtaken by a more stable innate predisposition to a naturalistic social object.

## Data Availability

The raw data supporting the conclusion of this article will be made available by the authors, without undue reservation.
