# Supplementary material for: The power of innate: Behavioural attachment and neural activity in responses to natural and artificial objects in filial imprinting in chicks
Source: Front Physiol. 2022 Nov 21;13:1006463. doi: 10.3389/fphys.2022.1006463 (PMC9720186; doi:10.3389/fphys.2022.1006463)
Supplement: Supplementary file 1 [file Table1.docx]

**Supplementary Table 1.** Number of chicks in experimental treatments

|  | **Imprinted to fowl** | **Imprinted to box** |
| --- | --- | --- |
| **Protocol 1 (“weak” training):** | **28** | **20** |
| “Weak” training and T1 test | 28 | 20 |
| ISH after training and T1 test | 9 | 6 |
| Used for T2 test | 12 | 12 |
| Excluded due to low activity | 7 | 2 |
| **Protocol 2 (“strong” training plus re-training):** | **19** | **11** |
| Re-training and T3 test | 19 | 11 |
| ISH after re-training and T3 test | 4 | 4 |
| Excluded due to low activity | - | - |
| **Protocol 3 (“strong” training plus**  **reverse training):** | **24** | **23** |
| Reverse training and T3 test | 24 | 23 |
| ISH after reverse training and T3 test | 7 | 6 |
| Used for T4 test | 9 | 9 |
| Excluded due to low activity | 8 | 3 |
| **Total number:** | **71** | **54** |
