# Supplementary figures and images for: The power of innate: Behavioural attachment and neural activity in responses to natural and artificial objects in filial imprinting in chicks
Source: Front Physiol. 2022 Nov 21;13:1006463. doi: 10.3389/fphys.2022.1006463 (PMC9720186; doi:10.3389/fphys.2022.1006463)

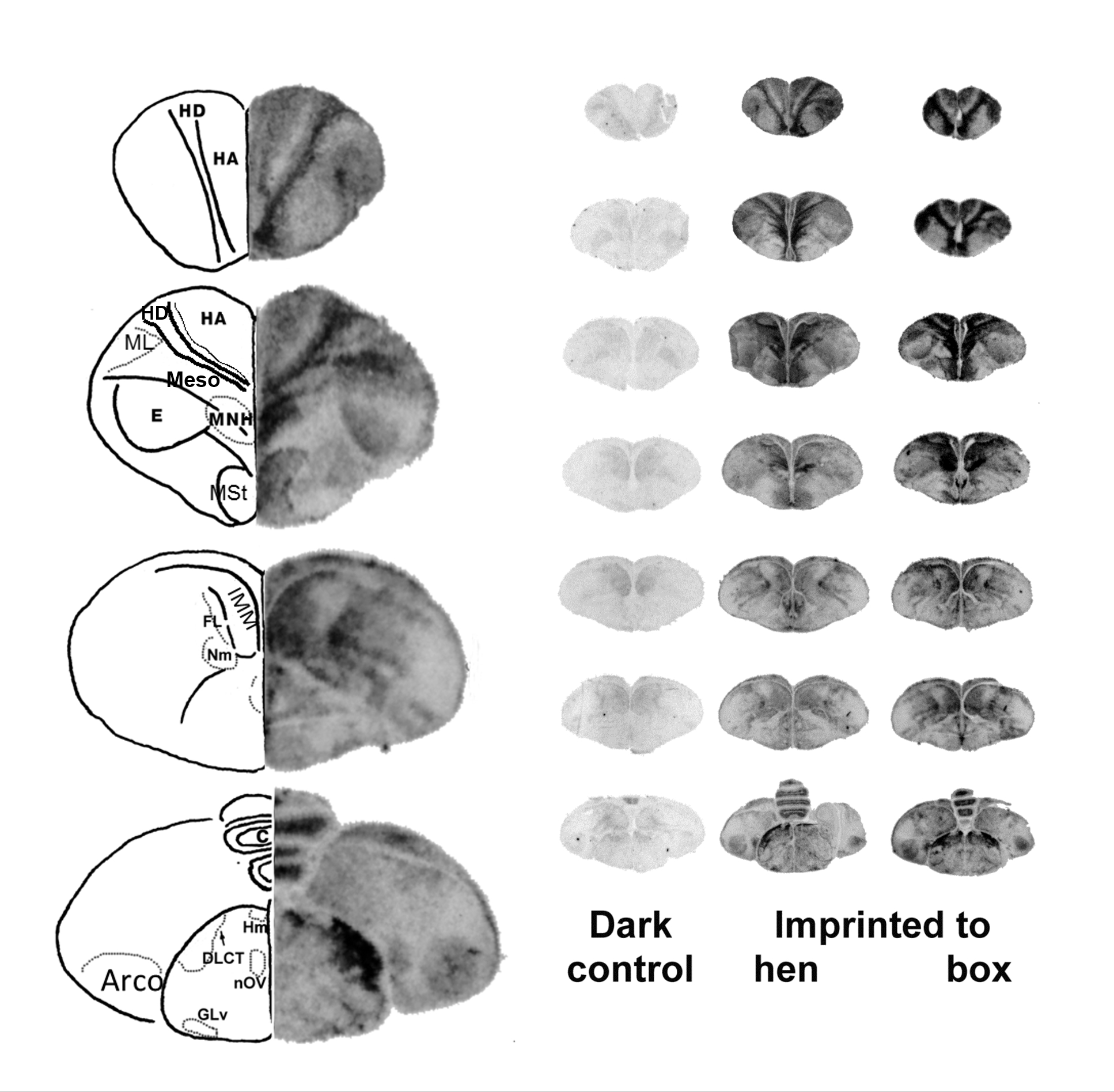

Supplement: Supplementary file 2 [file Image1.JPEG]
